# Obesity accelerates brain ageing: a multimodal imaging study

**DOI:** 10.1093/braincomms/fcaf389

**Published:** 2025-10-13

**Authors:** Federico Vanni, Sebastiano Cinetto, Michele De Filippo De Grazia, Marco Zorzi, Nicola Filippini

**Affiliations:** Laboratory of Neuroimaging and Neurodegeneration, IRCCS San Camillo Hospital, Venezia 30126, Italy; Department of General Psychology, and Padova Neuroscience Center (PNC), University of Padova, Padova 35131, Italy; Laboratory of Neuroimaging and Neurodegeneration, IRCCS San Camillo Hospital, Venezia 30126, Italy; Laboratory of Neuroimaging and Neurodegeneration, IRCCS San Camillo Hospital, Venezia 30126, Italy; Department of General Psychology, and Padova Neuroscience Center (PNC), University of Padova, Padova 35131, Italy; Laboratory of Neuroimaging and Neurodegeneration, IRCCS San Camillo Hospital, Venezia 30126, Italy

**Keywords:** obesity, BMI, brain age, neuroimaging, machine learning

## Abstract

Obesity is a global health concern, and it is thought to accelerate the normal ageing process. Obesity has also been linked to neurodegenerative processes, possibly as a manifestation of accelerated brain-ageing. In this cross-sectional study we combined multimodal neuroimaging data and machine learning techniques to assess the discrepancy between brain-based predicted age and chronological age, known as *brain age delta*, in obese participants and in normal-weighted individuals using a tight matching for age, gender and education across groups. Data were taken from the publicly available dataset ‘The Cambridge Centre for Ageing and Neuroscience (Cam-CAN)’ covering the adult lifespan (18–90 years old). Overall, brain age delta was greater in obese individuals for grey matter (GM) and functional connectivity (intra- and inter-network connectivity) measures. When considering the age-range, the difference between groups peaked in mid-age (40–60 years old) for GM, while for intra-network connectivity it was more marked in late age (60–90 years old). Overall, our results provide evidence to the hypothesis that obesity accelerates the brain ageing process, with the earliest effect already evident in the 40–60 age range. Earlier intervention on obesity might contribute to maintain a healthy brain potentially reducing the risk of developing late-life brain-related pathologies.

## Teaser

The impact of obesity on brain ageing was investigated using multimodal neuroimaging and machine learning. Accelerated brain ageing was observed in obese relative to normal-weight individuals, peaking in midlife for grey matter and after 60 for functional connectivity measures. Early interventions for obese to maintain brain health is warranted.

## Graphical Abstract

**Graphical Abstract:**
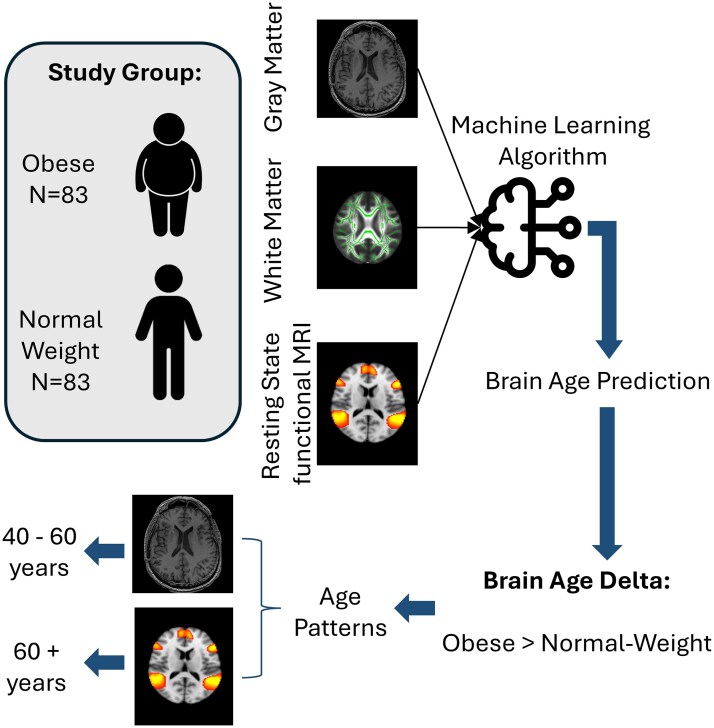

## Introduction

The last decades have seen an increase in life expectancy, with estimates suggesting that by 2050, there will be more than 2 billion people above 60 years of age.^1^ Increased longevity presents a valuable opportunity to remain productive and pursue one’s aspirations and passions, but this largely depends on maintaining and fostering good health. It is therefore crucial to promote the concept of healthy (‘successful’) ageing and to identify the factors that affect resilience against and vulnerability towards the development of late-life pathologies.

Obesity, which is defined as a body mass index (BMI) greater than 30 kg/m^2^, is rapidly becoming a major global health concern, affecting about 16% of the adult population (890 million individuals).^2,3^

The prevalence of adults with obesity has more than doubled in the last 40 years and, if this trend continues, it is estimated that by 2050, approximately one-third of the global adult population will be overweight or obese.^4-6^ From a medical perspective, obesity has been shown to be related to greater risk of some comorbid conditions, such as cerebrovascular disease, type 2 diabetes mellitus and hypertension, among others,^7^ but also lower cognitive performance and later in life dementia.^7^

Recent studies have shown that within the elderly population the prevalence of obesity has been on the rise, compared to two to three decades ago.^8^ For instance, in the United Kingdom, in the age range 65–74 years old, 30% of females and 33% of males are categorized as obese,^9^ whereas among individuals older than 75 years females and males with obesity are, respectively, 28% and 23%.^9^ Given these demographic shifts (the rising prevalence of elderly and of obesity) and the impact their comorbidities have on health, it has become fundamental studying these intertwined aspects and their interaction.

Up to date, several studies have investigated the connection between obesity and ageing, with the prevailing hypothesis that obesity might be linked to an accelerated ageing process.^10,11^ Studies in mice have shown that when obesity is present, the health consequences to comorbid conditions are similar to those found in a more advanced state of ageing.^11^ In addition, neurodegenerative processes seem to occur at a younger age in individuals with obesity relative to non-obese people.^12^ A similar interaction is also assumed to exist between obesity and brain-ageing, directly affecting brain morphology and/or functionality integrity.

Neuroimaging techniques have proved to be sensitive at assessing and identifying age-related brain changes before any cognitive symptom or impending disease. Magnetic Resonance Imaging (MRI) studies have consistently shown that ageing is associated with a reduction of grey matter (GM) volume, both on a global and a regional level.^13,14^ Additionally, resting-state functional MRI (rs-fMRI) analyses have revealed changes in the functional connectivity of intrinsic brain networks during ageing.^15,16^ Specifically, within-network (hereafter, intra-network) connectivity appears to decrease, whereas between-networks (hereafter, inter-network) connectivity seems to increase,^15,17-19^ despite some heterogeneity in findings.^18^ Notably, highly similar alterations in brain structure and function have been associated with increasing BMI. Indeed, neuroimaging studies have shown negative correlation between BMI and both global and regional GM volume, especially within motor, frontal and occipital areas.^7^ Moreover, individuals with obesity showed reduced intra-network connectivity associated with higher BMI, whereas inter-network connectivity changes associated with obesity have been scarcely investigated.^20^ Taken together, these results seem to suggest that obesity affects the brain in a similar way to the ageing process, potentially interacting with it and accelerating its progression. This has also been observed in a study by Ronan and colleagues, where obese individuals had brain features more closely resembling those of normal-weighted individuals who were significantly older (reaching the greatest difference between the two groups during mid-life).^12^

Here, we addressed the role played by obesity in modulating age-related brain changes by exploiting the ‘*brain-age*’ framework, which aims at predicting individual brain ageing based on advanced neuroimaging techniques combined with machine learning tools.^21^ This approach is increasingly being adopted to investigate how ageing may impact the brain, as well as to provide insights on the brain’s health status.^22-24^ Indeed, the discrepancy between brain-based predicted age and chronological age, known as *brain age delta*, is used to index the degree to which an individual deviates from healthy brain-ageing trajectories (e.g. accelerated brain ageing) and it provides a biomarker of an individual’s brain health.^21,24^

Multimodal neuroimaging data (including structural, diffusion and functional MRI) was taken from the Cambridge Centre for Ageing and Neuroscience (Cam-CAN) dataset,^25,26^ which includes a large cohort of participants covering the whole adult lifespan (18–89 years old). Within this dataset, we identified a sample of 83 individuals with BMI in the obesity range as well as an equally sized normal-weighted control group tightly matched for socio-demographic characteristics, which ensured that potential differences in brain age was not driven by uncontrolled variables.

Our aim was 3-fold: (i) quantify the brain age discrepancy between obese and normal-weighted individuals, (ii) identify which MRI-derived measure, as a proxy of brain ageing, was the most sensitive to this effect, (iii) determine the timeline of these changes. The hypothesis that brain ageing is accelerated by obesity leads to the straightforward prediction of higher brain age delta in the obese group compared to the normal-weighted group. In order to identify which type of MRI measure was most sensitive to the effect of the obesity status, brain age was independently predicted from measures of GM volume, white matter (WM) integrity and functional connectivity. Finally, we identified three age ranges (‘young’, 20–40 years old, ‘middle age’, 40–60 years old and ‘older’ 60+ years old) to explore at which specific age range this effect starts becoming manifest.

Understanding the effect of obesity on brain ageing, identifying the most sensitive imaging measures, and determining when this effect becomes evident are relevant for several reasons: (i) quantifying the impact of obesity on the ageing brain can help to recognize early warning signs, leading to targeted interventions that may slow down or even prevent the onset of late-life pathologies; (ii) from a public health perspective, as obesity rates are rising worldwide, strategies can be developed to promote healthier lifestyles and prevent obesity-related cognitive decline on a large scale; (iii) MRI-derived measures, combined with machine learning approaches, can be powerful tools to identify viable neuroimaging markers. These markers, reflecting vulnerability and/or resilience to lifestyle factors, could potentially be used to test the effectiveness of treatments.

## Materials and methods

### Study sample

All participants included in this study were taken from the publicly available dataset Cam-CAN (available at https://cam-can.mrc-cbu.cam.ac.uk/dataset/).^25,26^ Data collection was performed in accordance with the Declaration of Helsinki, approved by the local ethics committee and all participants provided written informed consent prior to data acquisition for the study. Here, we selected subjects included in Stage II (‘CC700’ phase) of the Cam-CAN dataset. Inclusion criteria comprised: (i) recorded information for socio-demographic (age, sex, education, handedness) and cognitive (mini-mental-state-examination, category and letter verbal fluency scores) variables, (ii) recorded information for height and weight, in order to calculate the BMI score [(cm)^2^/kg], (iii) availability of good quality structural, diffusion weighted imaging (DWI) and rs-fMRI scans.

We identified 535 participants across the entire lifespan (18–89 years old) who met the inclusion criteria. All participants had a normal Mini Mental State Examination (MMSE) score (≥25). The participants were divided based on their BMI scores: (i) Normal weight (NW), 18.5 ≤ BMI ≤ 24.9, (ii) Overweight, 25 ≤ BMI ≤ 29.9, (iii) Obese (O), BMI ≥ 30. The characteristics of the NW and O groups are summarized in Supplementary Table 1. Given that the O group was older compared to the NW and, as previously shown,^12,27^ O subjects tend to have lower educational level and cognitive score relative to NW, we algorithmically matched the two groups based on age, gender and education. For each participant within the O group, the individual in the NW group with the closest age (with a maximum age difference of 5 years), matching gender, and educational level differing by at most one unit was selected. Notably, if the age difference exceeded 5 years, the obese individual was not matched and was therefore excluded from the O group (in total *n* = 8 obese subjects were excluded from analysis). The iterative procedure identified a final group of 83 individuals for the O group and 83 individuals for the normal-weighted control (NWc) group that was tightly matched for socio-demographic characteristics, as well as for cognitive scores and brain volume (see Table 1). This tight matching is crucial to minimize the potential effect of nuisance variables that might inflate group differences in the neuroimaging data: for example, education level may be a confounding factor because it is both negatively correlated with BMI^28^ and a moderator of brain ageing trajectories mitigating neurocognitive decline.^29-31^ Importantly, these two groups (NWc and O) were retained as held-out data in our machine learning approach to brain-age prediction. That is, only the remaining individuals in the dataset (*n* = 361, which included both normal-weighted and overweight individuals) were used for feature extraction and to train the models, thereby preventing methodological issues that can plague brain-based predictive modeling.^32^ Our choice to include overweight individuals for training the brain-age models had the following motivations: (i) increase the size of the training set, which is important to prevent overfitting; (ii) increase the variability in the data to prevent excessive specialization (if not overfitting) on normal-weighted individuals. In this regard, including overweight individuals in model training could reduce the discrepancy between obese and normal-weighted individuals at testing, thereby working against (rather than in favour of) our study hypothesis.

**Table 1:** Sociodemographic features of the two study groups

	NWc (N = 83)	O (N = 83)	*P*
Socio-demographics			
Age, years	61.93 (± 16.39)	61.67 (±16.25)	0.92
Educational, qualification	5/7/4/16/51	8/5/6/21/43	0.60
Sex (N, % female)	35 (42%)	35 (42%)	1
Handedness	77.19 (±51.39)	84.33 (±42.25)	0.33
PAEE	42.92 (±19.34) [89%]	39.48 (±21.93) [87%]	0.31
Cognitive scores			
MMSE	28.75 (±1.33)	28.42 (±1.42)	0.13
Verbal fluency	17.44 (±6.15)	17.32 (±5.83)	0.89
Verbal category	23.21 (±6.26)	22.90 (±7.56)	0.77
Brain features			
Whole brain volume	1607.28 (±176.41)	1618.03 (±139.36)	0.66

Values denote mean (±SD), numbers of subjects or (percentage of available data). *T*-test and Chi-square were used to compare continuous and categorical variables of the two study groups. NW = normal-weighted; NWc = normal-weighted control group; O = obese. Education level is expressed as a categorical variable based on qualification level: no degree/O-GCSE levels or equivalent/A levels or equivalent/NVQ, HND, HNC or other professional qualification/CSE or university. PAEE, physical activity energy expenditure.

Estimated physical activity energy expenditure (PAEE) per week was also evaluated using the European Prospective Investigation into Cancer Study-Norfolk Physical Activity Questionnaire (EPIC-EP) self-reported questionnaire.^33^ These data were available in more than 85% of the participants included in the study. Similarly, information about dietary habits was gathered using a self-reported questionnaire and collected either as ‘number of pieces/cups per day’ or ‘how many times per week’ a certain type of food was consumed. As expected, we observed some dietary profile differences between the NW and O participants, but most of the categories did not significant differences. Here, we report mean and standard deviation and *P*-values for each of the category evaluated: (i) Vegetables (NW: 7.02 ± 4.19, O: 6.35 ± 4.00, *P* = 0.18); (ii) Fruit (NW: 3.14 ± 2.32, O: 3.22 ± 3.00, *P* = 0.79); (iii) Fish (NW: 3.08 ± 1.38, O: 3.41 ± 1.46, *P* = 0.03); (iv) Meat (NW: 6.99 ± 3.42, O: 7.91 ± 3.04, *P* < 0.01); (v) Cheese (NW: 3.22 ± 1.15, O: 2.97 ± 1.19, *P* = 0.06); (vi) Bread (NW: 13.12 ± 9.74, O: 12.42 ± 8.87, *P* = 0.55); (vii) Cereals (NW: 4.63 ± 2.79, O: 4.49 ± 2.97, *P* = 0.63); (viii) Salt (extra salt added to food) (NW: 1.70 ± 0.96, O: 1.95 ± 1.13, *P* = 0.04); (ix) Hot Drinks (tea and/or coffee) (NW: 4.58 ± 3.13, O: 5.25 ± 2.96, *P* = 0.07); (x) Water (NW: 3.65 ± 2.83, O: 3.03 ± 2.31, *P* = 0.07). A similar pattern was also observed for the 83 NWc and 83 O participants included in the test sets: (i) Vegetables (NWc: 6.80 ± 3.50, O: 6.52 ± 4.04, *P* = 0.62); (ii) Fruit (NWc: 3.62 ± 2.00, O: 3.31 ± 3.05, *P* = 0.45); (iii) Fish (NWc: 3.19 ± 1.35, O: 3.42 ± 1.49, *P* = 0.21); (iv) Meat (NWc: 5.95 ± 3.51, O: 7.87 ± 3.12, *P* < 0.01); (v) Cheese (NWc: 3.43 ± 1.16, O: 3.03 ± 1.20, *P* = 0.02); (vi) Bread (NWc: 13.43 ± 10.16, O: 12.73 ± 9.07, *P* = 0.64); (vii) Cereals (NWc: 4.96 ± 2.96, O: 4.72 ± 2.87, *P* = 0.55); (viii) Salt (extra salt added to food) (NWc: 1.63 ± 0.95, O: 1.91 ± 1.11, *P* = 0.09); (ix) Hot Drinks (tea and/or coffee) (NWc: 4.48 ± 2.18, O: 5.18 ± 2.96, *P* = 0.08); (x) Water (NWc: 3.30 ± 2.18, O: 3.07 ± 2.30, *P* = 0.57).

### MRI data acquisition

MRI data were acquired at the Medical Research Council-Cognition and Brain Sciences Unit using a Siemens 3 Tesla (3T) TIM trio System (Siemens Healthcare GmbH, Erlangen, Germany). The scans used in the current study encompassed anatomical, diffusion MRI and rs-fMRI scans. The anatomical scans were performed using a 3D T1-weighted magnetization prepared rapid gradient echo (MPRAGE) sequence [Repetition Time (TR) = 2250 ms; Echo Time (TE) = 2.99 ms; flip angle = 9°; Inversion Time (TI) = 900 ms; voxel size = 1 mm isotropic; GeneRalized Autocalibrating Partial Parallel Acquisition (GRAPPA) acceleration factor = 2]. The DWIs were obtained with a twice-refocused spin-echo sequence (30 diffusion gradient directions for each of two *b*-values: 1000 and 2000 s/mm^2^, three images acquired with a *b*-value of 0, TR = 9100 ms, TE = 104 ms, voxel size = 2 mm isotropic, 66 axial slices, number of averages = 1). The rs-fMRI images were obtained using a Gradient-Echo Echo-Planar Imaging (EPI) sequence (TR = 1970ms; TE = 30 ms; flip angle = 78°; voxel-size = 3 × 3 × 4.44 mm). During the rs-fMRI acquisition, participants were asked to rest with their eyes closed.

Further information about the MRI acquisition protocol can be obtained from the protocol papers in.^25,26^

### MRI data processing

Data analysis was carried out using FMRIB Software Library (FSL) tools^34^ and Mrtrix3 software^35^ for part of DWI image pre-processing.

#### Anatomical scans

Pre-processing steps for anatomical images included: (i) re-orienting images to the standard (MNI) template, (ii) bias field correction, (iii) brain extraction and (iv) brain tissues segmentation using FMRIB’s Automated Segmentation Tool (FAST) that allows producing maps and derive measures of total GM, WM, and cerebro-spinal fluid (CSF) for each individual subject.

Volumetric measures for each participant were extracted using the Harvard-Oxford atlas, which includes 48 region-of-interests (ROIs) covering the entire cortex. This parcellation was done in order to also investigate whether there was any specific brain region driving the potential group-related difference in the age-prediction score. The 48 ROIs in MNI standard space were registered into the participants’ native space and overlaid to the GM map, in order to ensure that only GM areas were used for our analyses. Each participant GM ROI’s volume was normalized by the respective total brain volume.

#### DWI scans

Noise-level estimation and denoising (*dwidenoise*), Gibbs ringing artefacts removal (*mrdegibbs*), and motion and eddy current-induced distortion correction (*dwifslpreproc*) were carried out for each subject.^35^ Maps of Fractional Anisotropy (FA), Mean Diffusivity (MD), Axial Diffusivity (AD) and Radial Diffusivity (RD), all indices of WM integrity, were produced using DTIFit, part of FMRIB’s Diffusion Toolbox, that fits a diffusion tensor model at each voxel. The FA, MD, AD and RD output images were used as input for Tract-Based Spatial Statistics, a voxel-wise approach for analysis of DWI-derived measures.^36^ All subjects’ data were aligned into a common space using nonlinear registration (FNIRT). As per tool processing steps, the mean FA image was generated and thinned to create a mean FA skeleton, which represents the centres of all tracts common to the group. Finally, each subject’s aligned DWI data were then projected onto this skeleton and merged into a single 4D file. The JHU DTI-based WM atlas (ICBM-DTI-81), which includes 48 ROIs, was used to extract measures of WM integrity for FA, MD, AD and RD for each individual participant from the skeletonised 4D file.

#### Resting fMRI (rs-fMRI) scans

Data pre-processing included motion correction, brain extraction, Gaussian kernel smoothing of FWHM of 5 mm, high-pass temporal filtering with a cut-off of 100 s (0.01 Hz), and it was performed using first-level fMRI Expert Analysis Tool (FEAT) v. 6.00.^37^ FMRI volumes were registered to the individual’s structural scan and standard space images using both linear (FLIRT) and nonlinear (FNIRT) registration tools, then optimized using boundary-based-registration approach.^38^ FMRIB’s ICA-based X-noiseifier (FIX)^39^ was applied to denoise functional images from the spurious signal and increase the possibility of identifying markers of effective connectivity. A training dataset specifically developed on the Cam-CAN dataset was created. The best threshold for FIX in our dataset, to denoise functional images and remove motion-, physiological- and non-GM-related signal, was identified as 10 with a mean (median) true positive rate (TPR) and true negative rate (TNR) of 97.2% (100%) and 85.5% (87%), respectively, which is in line or above the suggested cut-offs (TPR > 95%—TNR > 70%).

A previously published set of 169 ROIs (hereafter, nodes) belonging to 10 well-established Resting State Networks (hereafter RSNs: visual-fovea and visual-periphery, sensory-motor, auditory, dorsal attention, ventral attention, language, fronto-parietal, cingulo-opercular, and default mode network)^40^ was used to extract the time-series from each participant’s rs-fMRI sequence. All voxels included in a 5 mm sphere centred on the ROI’s MNI coordinates were averaged to compute the node’s time-series. Two ROIs were discarded because they fell outside the brain mask. Whole-brain functional connectivity was then computed by correlating the time series for each pair of nodes. We then generated the intra-network connectivity matrix by selecting all pairs of nodes within each RSN (i.e. 1539 values). Conversely, the inter-network connectivity matrix was generated by selecting the functional connections between RSNs, that is all pairs formed by nodes belonging to two different networks (i.e. 12 322 values).

### Machine learning and statistical analyses

Machine learning analyses are detailed in the following subsections. Regularized regression was used for brain age prediction, whereas logistic regression was used for brain-based discrimination of obesity status. Moreover, functional connectivity underwent dimensionality reduction based on Principal Component Analysis (PCA) to extract a more compact set of features that were used as input for machine learning.

### Principal component analysis

PCA consists of a linear transformation of the original data into a smaller set of uncorrelated features that maximally explain the variability of the dataset.^41^ When applied to high-dimensional data like functional connectivity matrices, PCA returns a compact set of principal components (PCs) that can be used as features for machine learning-based brain-behaviour mapping.^42,43^ PCA was run separately on inter-network and intra-network connectivity matrices using the *PCA* class provided by *scikit-learn*, an open-source Python library for machine learning. The number of PCs to be retained as predictors was treated as a hyperparameter and optimized during regression. For intra-network connectivity, this parameter was selected from a range of values linearly spaced between 5 and 210, with a step size of 5, where 212 corresponds to the number of PCs explaining 95% of the variance. For inter-network connectivity, the range was extended to values between 5 and 245, still with a step size of 5, where 247 corresponds to the number of PCs explaining 95% of the variance.

### Regularized regression for brain-age prediction

Brain-age prediction was independently performed for each imaging modality (structural, diffusion, and functional MRI data) and for each of the derived measures. The features (regressors) entered in the different models (following standardization) were the following: (i) for structural MRI, the GM volume of the 48 ROIs normalized by total brain volume; for diffusion MRI, we assessed FA, MD, AD and RD as measures of WM integrity, using 48 ROIs; (iii) for fMRI, the PCs extracted from inter-network and intra-network connectivity matrices.

Prediction of brain age was based on elastic-net regression,^44^ which combines LASSO (L1) and ridge (L2) regularization to limit model complexity and prevent overfitting, thereby helping maintain the model more interpretable.^45^ Regularization is controlled by two hyperparameters, *α* and *λ*, and it can range from L1-based (when *α* = 1), which imposes exact zero values on coefficient estimates, to L2-based (*α* ≈ 0), which shrinks the coefficients to values near to zero, or to a combination of these (0 < *α* < 1). Moreover, a larger penalization strength (*λ*) results in more coefficients being reduced to zero. ElasticNetCV from the scikit-learn library was employed to fit the model. This estimator integrates cross-validation to automatically identify the best *α* and *λ* from a specified list of values. The *α* was selected from a range including 0.001, 0.25, 0.5, 0.75 and 0.99 while *λ* was chosen among 100 values logarithmically distributed between 10^−5^ and 10^5^. Hyperparameter optimization was performed using a 10-fold cross-validation scheme stratified by age (*StratifiedKFold* function from *scikit-learn*) to ensure that the training and test datasets were proportionally similar with respect to age. During each iteration, 1-fold was held out (validation set), while the others were used to estimate the best parameters of the model for every combination of hyperparameters. Subsequently, each model was evaluated on the validation set, using the mean squared error (MSE) between real age and predicted age. This process was repeated for all the 10 folds. The combination of hyperparameters with the lowest MSE was selected and the model was then fit again on the entire training sample. Note that the training and cross-validation procedure did not include individuals in the O and NWc samples, which were treated as held-out data and only used for testing to collect predicted brain-age. We also carried out permutation testing to assess whether model performance reflected a genuine relationship between the input features and brain age. In this procedure, the target variable (true age) was shuffled, thereby disrupting any systematic relationship between predictors (GM, WM integrity measures, or connectivity measures) and age. We trained 1000 models on permuted datasets and computed Mean Absolute Error (MAE) on the held-out data to generate a null distribution. We reported the *P*-value obtained from comparing performance of the original model to the null distribution. Predicted brain age (as well as brain age delta) typically shows overestimation in younger individuals and underestimation in older individuals, which is caused by dilution bias in regression analysis.^24,46^ Removing this age-bias is important to ensure that any further analyses are not influenced by the age-dependence of the predictions. The conventional approach is to apply a statistical correction by fitting a regression model on predicted age as a function of chronological age.^24^ The slope and intercept of the regression model are then used to compute the corrected predicted age, which in turn is used to derive corrected delta values (i.e. corrected brain age delta = corrected predicted age − chronological age).^46^ Predictive performance was assessed on the latter index using the coefficient of determination (*R*²) and the MAE.

### External validation of the brain age model

We used a previously developed deep learning-based brain age prediction model^47^ to evaluate the robustness of our findings. The deep learning model receives a T1-weighted skull-stripped MNI-normalized structural image as input to return the estimated brain age and it was trained on a large-scale dataset (*n* = 11 729) covering individuals of ages 3–95 years collected from multiple studies.^47^ We used the model out-of-the-box to collect brain age predictions for our sample and then computed the brain age deltas. This ensured that any difference in brain age delta between O and NWc groups is not based on idiosyncratic characteristics of our training set, thereby providing an external validation of our findings.

### Brain-based classification of obesity status

MRI-derived measures yielding significant differences in age prediction error (*brain age delta*) between O and NWc were also used to train models for classification of the obesity status. In this second part, the number of PCs used for intra- and inter-connectivity corresponded to those explaining 95% of the variance in the training set. We used logistic regression as classifier, provided by the *LogisticRegression* class in scikit-learn. In addition to MRI-derived measures, age and gender were used as predictors for the model. The dataset was split into 10 folds with a *k*-fold cross-validation scheme stratified by age. During each iteration, one group was held out (test set), while the others were used to fit the model and to tune the hyperparameters, namely the tolerance, that was selected from a range including 3, 2, 1, 0.5, 0.1, 0.05, 0.01, 0.001, 0.005, 0.001 and 0.0001, and C, that was chosen among 30 values logarithmically distributed between 10^−4^ and 10^4^. A ridge regularization was used to limit model complexity and prevent overfitting. Subsequently, the model was evaluated on the test set, and predictions (O or NWc) were recorded. This process was repeated for all the 10 folds, which ensures that predictions were generated for all individuals within the dataset. Area under the curve (AUC), sensitivity (SE) and specificity (SP) were utilized to evaluate the predictive performance of the different models. To ensure more stable results, this process was repeated 10 times using *RepeatedStratifiedKFold* function from *scikit-learn*, and the mean scores for each metric were calculated.

### Bootstrap testing

Bootstrap testing was used to assess feature stability for both age prediction and obesity status classification (obese vs. normal weight), across all measures (GM, WM integrity measures and connectivity measures). After the best combination of hyperparameters was selected during cross-validation, a dataset of the same size as the training set was sampled with replacement. A new model was then trained on this resampled training set, and this process was repeated for 1000 iterations. Confidence intervals for the feature importance were computed based on the 2.5th and 97.5th percentiles of the resulting distribution.

### Statistical analyses

*T*-test and Chi-square were used to compare continuous and categorical socio-demographic variables of the two study groups. Brain age delta computed for the held-out NWc and O samples (for each model) were compared using a two-tailed *T*-test. Furthermore, when the difference between groups was statistically significant, we exploratively assessed whether this effect was most evident in a specific age range. We identified three distinct age ranges: 18–40 years old (‘younger’), 40–60 years old (‘mid-age’), and over 60 years old (‘older’). The rationale for this stratification was 2-fold: (i) brain volume tends to remain stable up to 40 years old, after which the process of brain atrophy becomes more pronounced,^48^ whereas age-related pathologies and comorbidities often tend to emerge and become more common around 60 years of age^49^; (ii) a prior study suggests that the most significant effect of obesity on brain age occurs during mid-age.^12^ Moreover, similar subgroups have been previously used to study changes in resting-state intra- and inter-network connectivity across the lifespan.^50^ For each of the three group ranges identified we therefore compared the subgroups of NWc and O participants with a two-tailed *T*-test.

Statistical analyses were performed using Python and SPSS software (SPSS, Inc. version 28).

## Results

### Grey matter analysis

**Figure 1:**
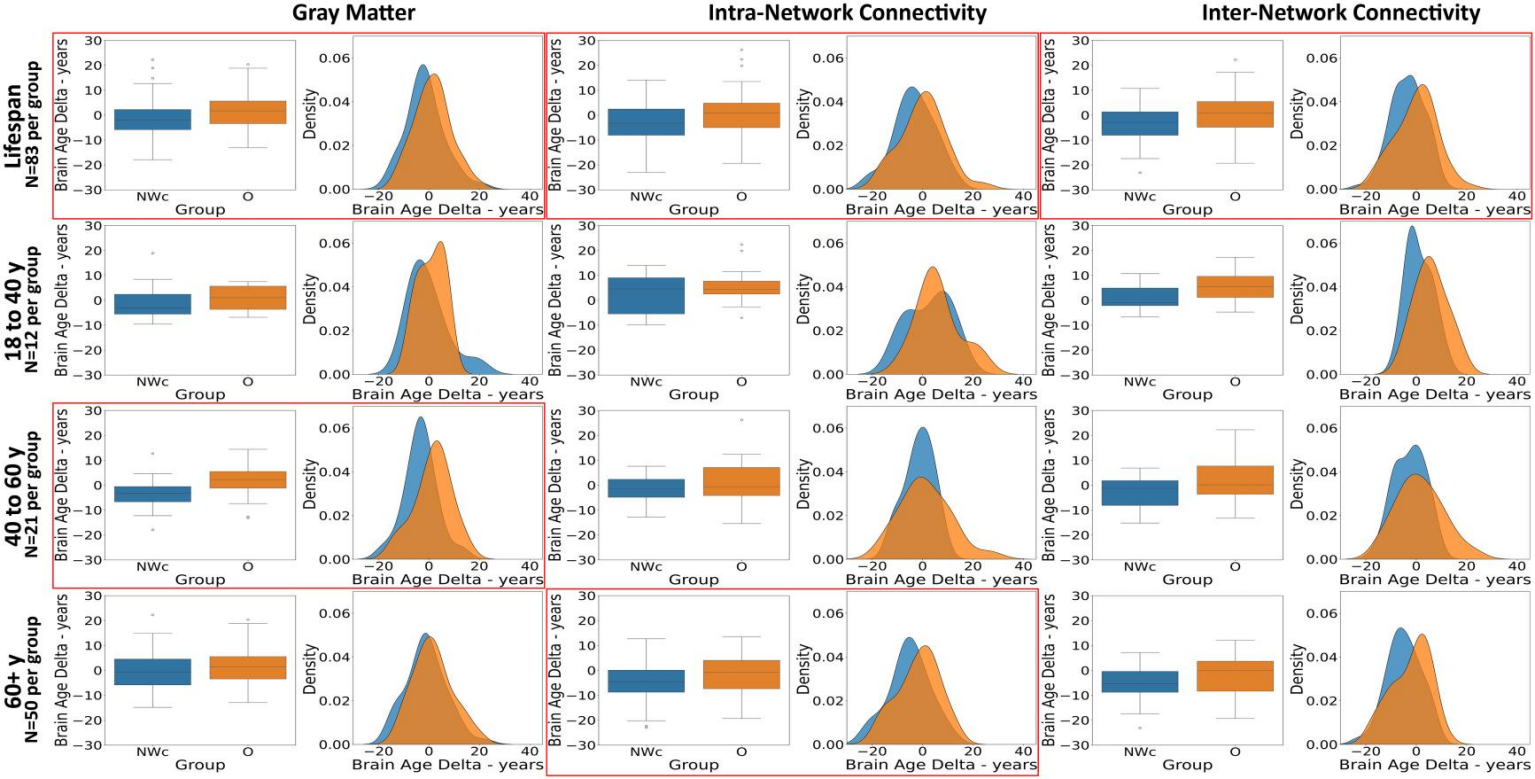
**Boxplots and density (reflecting the proportions of participants) plots of corrected brain age deltas for the two study groups for the entire lifespan and across different age ranges (18–40 years old, 40–60 years old, 60+ years old) and brain modalities (GM, between-network connectivity, within-network connectivity).** Significant differences between the two groups (*P* < 0.05, two-tailed *T*-test) are indicated by frames. NWc = normal-weighted control, O = Obese. *n* = number of participants included for each of the two study groups across the lifespan and for each of the three individual age ranges. y = years old. Single data points depicted in the Box Plots represent individual participants with values exceeding ±2 SD from the mean.

GM volumes for all ROIs were entered as regressors in the age prediction model. The model was fitted on the training set (hyperparameter tuning returned *α* = 0.001 and *λ* = 0.002) and subsequently tested on held-out test sets (O and NWc groups). Permutation testing showed that our model’s prediction performance was significant against the null model distribution (*P* < 0.001, 1000 permutations). The model’s predictive accuracy on the NWc group (*R*^2^ = 0.78, MAE = 5.98) and on the O group (*R*^2^ = 0.79, MAE = 5.87) was aligned with those reported in classic brain-age prediction studies.^21,51^ Comparison of the brain age deltas between NWc and O revealed a significant difference (*P* = 0.01), with the O group showing a greater brain age delta (average difference of 3 years) compared with the NWc group (see Table 2 and Fig. 1). When exploring the group difference in the three age ranges, the effect was mostly reliable in the mid-age window (*P*  *=* 0.02) with a deviation of nearly 5 years, and it showed a trend in the older but not in the younger participants.

**Table 2:** Brain age deltas of the two study groups is reported for the whole lifespan and across different age ranges (18–40 years old, 40–60 years old, 60+ years old) and brain modalities (GM, between-network connectivity, within-network connectivity)

	NWc (N = 83)	O (N = 83)	*t*	*P*	*Δ(O-NWc)*
Grey matter (lifespan)	−1.60 (±7.60)	1.41 (±7.24)	**2**.**613**	**0**.**01**	3.01
18–40 years old (N per group = 12)	−0.33 (±7.57)	1.01 (±5.23)	0.483	0.63	1.34
40–60 years old (N per group = 21)	−3.42 (±6.44)	1.37 (±7.01)	**2**.**250**	**0**.**02**	4.79
60+ years old (N per group = 50)	−1.14 (±7.84)	1.52 (±7.66)	1.700	0.09	2.66
Inter-network connectivity (lifespan)	−3.55 (±7.14)	−0.38 (±8.62)	**2**.**57**	**0**.**01**	3.16
18–40 years old	1.98 (±4.64)	6.7 (±6.05)	2.055	0.05	4.72
40–60 years old	−2.36 (±6.3)	1.08 (±8.9)	1.414	0.17	3.45
60+ years old	−5.37 (±7.1)	−2.7 (±7.85)	1.77	0.08	2.67
Intra-network connectivity (lifespan)	−3.07 (±8.02)	0.23 (±9.04)	**2**.**49**	**0**.**01**	3.30
18–40 years old	3.10 (±7.74)	6.06 (±7.88)	0.887	0.385	2.95
40–60 years old	−1.86 (±5.80)	0.94 (±9.58)	1.121	0.27	2.81
60+ years old	−5.07 (±7.95)	−1.47 (±8.33)	**2**.**186**	**0**.**03**	3.60

*T*-test was used to compare the two study groups. Significant differences between the two study groups are highlighted in bold.

### External validation with an independent brain age model

Our key findings were fully replicated using an independent deep learning model that was trained on a large-scale dataset to predict brain age directly from the T1-weighted structural scans. When applied to our sample of participants, the O group exhibited a significantly higher brain age delta compared to the NWc group, with an overall mean difference of approximately 3 years (*P* = 0.002). Additionally, when considering the three age ranges, this difference was significant only in the 40–60 age range, where the brain age delta in the O group exceeded that of the NWc group by an average of 5 years (*P* = 0.004).

### White matter analysis

FA, MD, AD and RD measures for all ROIs were independently entered as regressors for age prediction. Following tuning, the model’s hyperparameters were determined as follows: FA (*α* = 0.250 and *λ* = 0.008); MD (*α* = 0.008 and *λ* = 0.001); AD (*α* = 0.007 and *λ* = 0.250) and RD (*α* = 0.007 and *λ* = 0.001). Finally, each model was tested on the held-out NWc and O groups. Permutation testing showed that the predictive performance of the four models was significant when tested against the respective null distributions (*P* < 0.001 for all the four measures, 1000 permutations). The performance of the models on the NWc and O groups across the four WM measures ranged between 0.59 and 0.72 for the *R*^2^ and 6.6 and 8.24 for the MAE [FA (NWc: *R*^2^ = 0.76, MAE = 6.60, O: *R*^2^ = 0.67, MAE = 6.61), MD (NWc: *R*^2^ = 0.64, MAE = 7.86, O: *R*^2^ = 0.61, MAE = 7.85), AD (NWc: *R*^2^ = 0.72, MAE = 7.03, O: *R*^2^ = 0.59, MAE = 8.24), RD (NWc: *R*^2^ = 0.72, MAE = 6.96, O: *R*^2^ = 0.66, MAE = 7.40)]. However, no significant overall differences for any of the WM measures were found when brain age delta were compared between the two study groups (FA: *P* = 0.11; MD: *P* = 0.98, AD: *P* = 0.12; RD: *P* = 0.16).

### Functional connectivity analysis

For intra-network connectivity, hyperparameter tuning during training yielded the selection of 185 PCs, *α* = 0.001 and *λ* = 0.351. For inter-network connectivity, the number of PCs selected during training was 205, with *α* = 0.25 and *λ* = 0.068. Subsequently, both models were tested on the held-out NWc and O groups. Permutation testing showed that the predictive performance of the two models were significant when tested against the respective null distributions (*P* < 0.001 for both measures, 1000 permutations). Predictive performance ranged between 0.69 and 0.77 for *R*^2^, with MAE ranging between 6.32 and 7.00 (NWc group: intra-network, *R*^2^ = 0.72, MAE = 6.83; inter-network, *R*^2^ = 0.77, MAE = 6.32; O group: intra-network, *R*^2^ = 0.69, MAE = 7.00; inter-network, *R*^2^ = 0.72, MAE = 6.81).

As shown in Table 2 and Fig. 1, the brain age delta was overall (across the lifespan) significantly greater in the O group relative to the NWc group, for both intra- and inter-network connectivity measures, with an average difference of 3.30 and 3.16 years (*P*  *=* 0.01 in both cases), respectively. Follow-up exploratory analyses on the three different age ranges revealed that the dissimilarity between groups for intra-network connectivity was reliable only for the ‘older’ participants, with the O group showing a maximum deviation of 3.6 years (*P*  *=* 0.03) relative to the NWc group.

### Brain-based classification of obesity status

**Figure 2:**
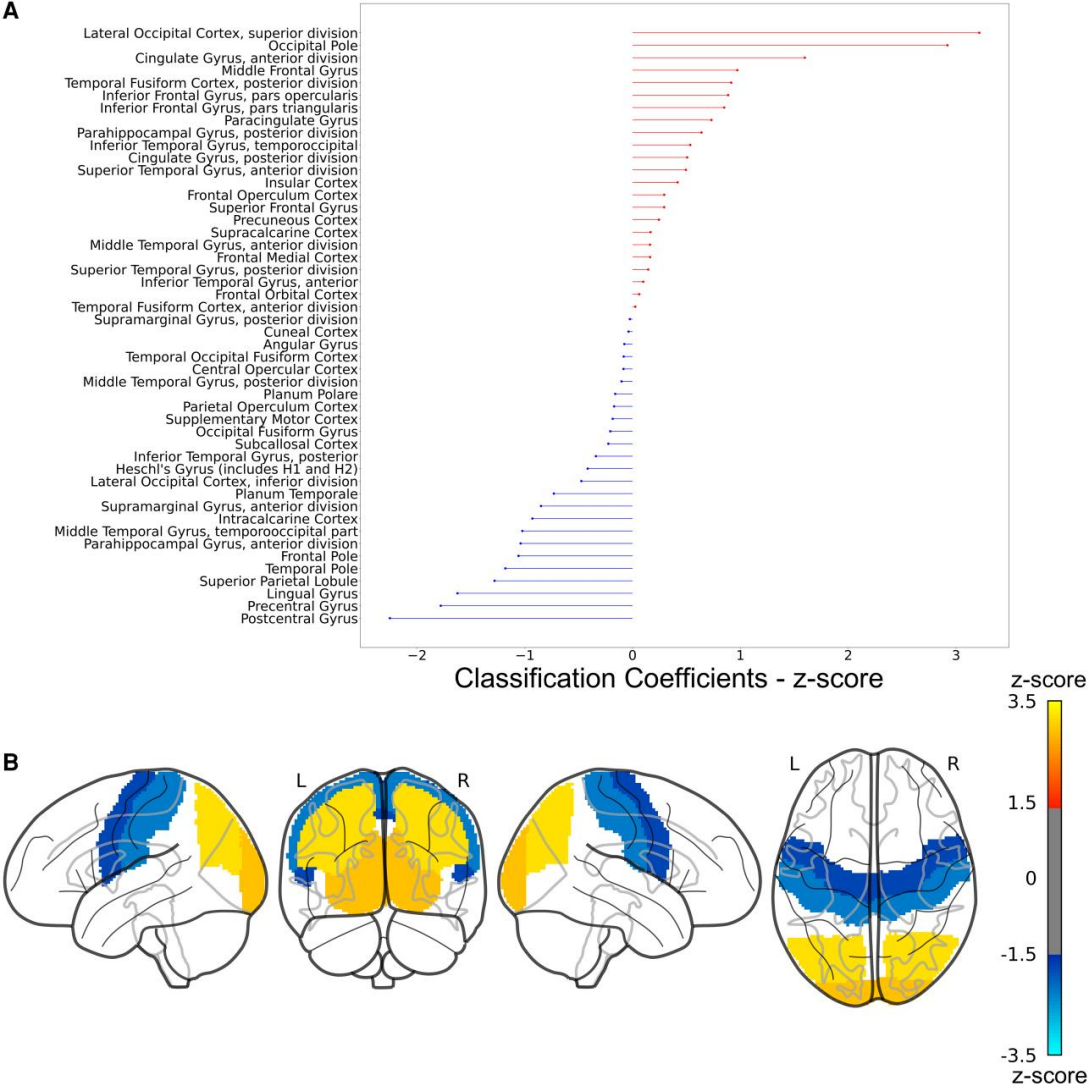
**(A) standardized classifier weights of the logistic regression model sorted by value.** Negative coefficients associate lower GM volume with increasing likelihood of predicting an obese condition, whereas positive coefficients indicate the opposite relation. (**B**) Brain regions with *Z*-score>±1.5 for the standardized classifier weights and surviving bootstrap correction are projected on a glass standard brain and colour-coded accordingly (*Z*-score < −1.5 blue to light-blue; *Z*-score > 1.5 red to yellow). L = left hemisphere, R = right hemisphere.

GM volumes, intra-network connectivity and inter-network connectivity measures, showing significant differences in the brain age delta between the two groups, were used to train classifiers that had to distinguish between O and NWc participants [hyperparameter tuning returned for GM: *C* = 1.29(±1.16) and tolerance = 0.011(±0.01); for intra-network connectivity: *C* = 0.01(±0.02) and tolerance = 0.03(±0.039); for inter-network connectivity: *C* = 0.01(±0.01) and tolerance = 0.09(±0.02)]. When gauged in terms of AUC, the cross-validated predictive performance was moderate for the GM-based model [AUC = 0.69(±0.01), SP = 0.71(±0.04), SE = 0.64(±0.05)] and poor for the models based on functional connectivity [intra-network connectivity: AUC = 0.58(±0.02), SP = 0.79(±0.09), SE = 0.41(±0.08); inter-network connectivity: AUC = 0.61(±0.01), SP = 0.66(±0.10), SE = 0.58(±0.11)]. Nevertheless, we examined the performance of the models within the specific age ranges where significant differences in brain age delta between the O and NWc groups were observed. Notably, the GM-trained model showed excellent performance in the mid-age age with AUC of 0.87(±0.02) [SP = 0.90(±0.01); SE = 0.83(±0.03)]. In contrast, the model trained on intra-network connectivity remained poor even in the older age range [AUC = 0.59(±0.02); SP = 0.78(±0.07); SE = 0.49(±0.07)]. The lower discriminant power found with functional measures relative to GM measure, even in the significant age-ranges, may reflect the higher variability observed in functionally derived measures.^24^ For the GM-based model, we inspected the classifier weights and ranked them according to their standardized values to determine which ROIs were most influential in predicting the obesity status. The weight values are plotted in Fig. 2 (panel A), with the largest weights (*Z*-score>±1.5) also projected on a glass brain (panel B). A negative weight indicates that larger ROI volume will increase the likelihood of predicting a normal weight condition, whereas smaller volume will increase the likelihood of predicting an obese condition. The most influential ROIs with negative weights were post-central gyrus, pre-central gyrus and frontal pole. Conversely, a positive weight indicates that increasing ROI volume is associated with higher likelihood of predicting an obese condition. This was the case of brain areas localized in the Occipital lobe. Bootstrap analysis was performed to assess the stability of these ROIs in order to determine whether these features were consistently important across resampled datasets. Notably, the ROIs that demonstrated greater stability overlapped with those identified as most influential in the model, including Postcentral Gyrus, Precentral Gyrus, and Occipital regions, with the exception of the Frontal Pole. The average standardized coefficients for these areas across bootstrap samples exceeded the 1.5 standard deviation threshold. Additional ROIs exhibiting high stability included the Anterior Cingulate, the triangular part of the Inferior Frontal Gyrus, Lingual Gyrus, anterior and posterior Parahippocampal Gyri, Planum Temporale, anterior Superior Temporal Gyrus, and the anterior Temporal Fusiform Cortex. However, the average coefficient values for these regions across the bootstrap iterations did not surpass the 1.5 SD threshold.

## Discussion

The main aim of the current study was to test the hypothesis that obese participants show accelerated brain ageing in comparison to a matched control group of normal-weighted healthy individuals. Here, we examined the brain age delta, that is the discrepancy between brain-based predicted age and real chronological age, obtained from machine learning models trained on structural MRI (GM volume), diffusion MRI (WM integrity) and resting-state functional MRI (functional connectivity). The analyses allowed us to identify which MRI-derived measure can reliably differentiate the two groups. We then explored whether this difference was more pronounced in a specific age-range (in particular, mid- versus late-age ranges) and used multivariate classifiers to identify the brain regions that show greater contribution to discriminating the obesity status.

### BMI influences age prediction on morphological measures

A larger unaffected brain volume has been hypothesized to be a crucial factor associated with brain health.^52^ Recently, DeCarli and colleagues reported an increase of brain volume over decades of birth from 1930 to 1970,^52^ suggesting that these findings probably reflected improvements in early life environmental influences, such as improvements of health, socio-cultural conditions, and education,^52^ potentially protecting the brain against the risk for late-life dementia. Indeed, accelerated loss of GM and WM have been often associated with cognitive decline^53-55^ and they may represent an indicator of underlying pathology.^52,56^ Our findings suggest that the O group had a significantly higher brain age delta compared to the NWc group overall, implying that obesity might be associated with a faster age-related GM reduction, as previously hypothesized.^57^ We found that this disparity was mostly pronounced in the ‘mid-age’ range, where the average difference between the two groups reached a peak of 5 years. Such accelerated GM loss may represent an initial threat to the brain integrity making it more vulnerable and less resilient to effectively counteract the ageing process and therefore potentially heightening the risk of developing future pathologies.^12^

Conversely, the difference in brain age delta between the two groups was not evident in the other two age ranges. In the younger age group, this could be attributed to the time required for the effects of obesity on the brain to become noticeable and significant, along with a potentially lower statistical power due to a smaller sample size. In the older age group, the more pronounced difference observed in the middle age may gradually wear itself out due to the physiological age-related brain atrophy becoming more evident for the NWc group.^48^ Nevertheless we noticed a trend towards group difference also in this age-range, which could suggest a type-II error caused by the small sample size. Importantly, an independent deep learning model for age prediction,^47^ trained on a large-scale dataset of T1-weighted images produced similar results when applied to our study groups, thereby providing additional support for the robustness and potential generalizability of our findings.

The brain-based classification of obesity status analysis revealed that the brain regions mostly driving the observed effect were the post- and pre-central gyri (O < NWc), and brain areas localized in the occipital lobe (O > NWc). These results are in line with previously published imaging studies^7,58,59^ reporting volume reduction in O relative to NW individuals in pre- and post-central brain regions and increased brain volume in O relative to NW mostly localized in the occipital lobe. The regions showing the greatest sensitivity to obesity could potentially be used as markers to evaluate the efficacy of future treatments.

No significant differences were observed between the two groups for WM measures. Several studies have similarly reported no significant difference in WM integrity,^60-62^ although other studies have reported reduced WM integrity associated with obesity,^63,64^ suggesting an inconsistency in findings as reported by Daoust and colleagues in a recent meta-analysis.^65^

### BMI influences age prediction on functional connectivity measures

For the resting state functional connectivity analysis, we derived intra-network and inter-network connectivity measures. Intra-network connectivity reflects the strength of connectivity within each specific network, whereas inter-network connectivity refers to the strength of interaction between networks. In both cases, the O group had overall significantly greater brain age delta relative to the NWc group, indicating that obesity might also influence brain cytoarchitectonic functional connectivity, with inter- and intra-network connectivity patterns resembling those observed in participants older than their chronological age. Consistent with these findings, other studies reported reduced within networks connectivity in individuals with obesity,^20,66,67^ mirroring patterns of functional connectivity commonly seen in elderly individuals. Unfortunately, to our knowledge, there is no evidence for inter-network connectivity changes associated with obesity. Interestingly, studies on functional connectivity covering the adult lifespan have found a nonlinear trajectory for both intra- and inter-network connectivity measures.^15^ In details, intra-network connectivity seems to have a negative quadratic (inverted U-shape) trajectory, starting to decline around 50–60 years.^68^ Conversely, inter-network connectivity shows a positive quadratic (U-shape) trajectory from around 20–80 years, with the lowest level of connectivity reached in the fourth decade followed by an increase which becomes more evident in the sixth decade of life.^69^ Results reported in our study seems to suggest that the BMI may impact the decline process of the intra-network connectivity measure even further than physiologically expected (i.e. greater brain age delta in the O group relative to the NWc group in the ‘older’ range of age).

### Physiological interpretation

Obesity has been associated with a series of physiological changes that can affect neuronal integrity potentially explaining our results. Indeed, obesity has been linked to a state of low-grade chronic inflammation,^70^ due to the release of inflammatory cytokines, which can contribute to the degeneration of brain cells^71^ and damage the blood brain barrier,^72^ therefore limiting its protective role from harmful substances.^72,73^ Another common feature of obesity is insulin resistance,^74^ which causes higher levels of circulating insulin and glucose, potentially leading to brain damage and reduced neuronal maintenance over time.^75^ Moreover, obesity has been shown to be related to great levels of oxidative stress resulting in brain cells damage and accelerated neurodegenerative processes.^76,77^ Furthermore, obesity is a known risk factor for cardiovascular diseases,^78^ which in turn can negatively affect brain blood flow causing brain cells to become oxygen-deprived and also contributing to the accumulation of plaques and narrowing of blood vessels, further compromising brain health.^79^ The combination of mechanisms/factors like inflammation, insulin resistance, oxidative stress and cardiovascular issues, can over time create an environment which may hinder the brain’s ability to repair itself and form new neural pathways leading to accelerated brain ageing. Identifying when these mechanisms start becoming irreversibly deleterious and whether it is possible to stop/reverse their effect it would be also crucial for maintaining/restoring a person’s brain health.

### Limitations and future research

Limitations have to be considered in interpreting our findings. Firstly, sub-division of the two testing groups (NWc and O) based on the age-group status (‘younger’, ‘middle-age’ and ‘older’) led to a limited number of available participants per group, particularly in the ‘younger’ age-range. Future studies should replicate our findings using a larger sample of obese participants (and matched controls). Secondly, here we employed a static value for the BMI index, measured at a single timepoint. It would be interesting to investigate dynamic BMI changes over time and how these interact with brain-derived measures. For instance, observing the impact of weight loss on potential changes in brain-derived measures would be highly valuable from an intervention perspective. Thirdly, more recently developed MRI sequences are now employed in large scale datasets to derive finer brain-related matrices compared the ones employed here. For example, in the case of diffusion data, MRI sequences with greater number of diffusive directions and multi-shell parameters would allow for the computation of WM-measures, such as neurite density and orientation density that might be more sensitive than FA to the effect played by BMI on WM integrity. Fourthly, physical activity and dietary habits were measured using self-reported questionnaires. This could potentially lead to biases in the derived measures.^80,81^ Further research into these variables using more objective measures warrants future investigation. Similarly, the potential role of genetic predisposition to obesity (genetics data were not available for this study) should be thoroughly explored. Finally, BMI has been criticized because it represents a coarse index to categorize participants into a specific group not taking into account specific factors, such as adiposity, fat distribution, muscle mass or bone density.^12^ However, in this study, we compared groups of participants at the extreme of the BMI continuum (i.e. normal weighted and obese) and this could attenuate the potential mis-categorization of participants due to the factors mentioned above. Nevertheless, it would be useful to investigate whether measures more closely reflecting adiposity, fat distribution, muscle mass or bone density may be more sensitive and accurate in predicting individual brain ageing. Last but not least, the data used in this study are almost entirely derived from UK participants, which suggests that the generalizability of our findings has to be carefully weighed.

## Conclusions

In this study we provided support to the hypothesis that obesity might accelerate the ageing of the brain, potentially rendering it more vulnerable to late life insults and less able to cope with the ageing process. Here, indeed, we observed that obesity was associated with structural and functional alterations of the brain across the lifespan, with specific effects in well-defined age-ranges. Indeed, these changes do not seem to manifest uniformly across different stages of the lifespan or across different brain modalities. Specifically, we observed more pronounced alterations during mid-age, particularly in GM, whilst some others (i.e. functional intra-network connectivity) seem more prominent in the older age-range. Notably, our findings suggest that it could be already crucial intervening on obesity during the mid-age phase of life, which is not merely a transitional phase to elderly life, but it emerges as a critical period characterized by a multitude of processes that significantly influence future cognitive trajectories and brain health.^82^ Indeed, weight loss interventions, such as diet, exercise, or in extreme cases bariatric surgery, have been shown to mitigate functional connectivity changes and brain volume loss associated with obesity^83^ maintaining and/or restoring an adequate brain health status, which is a key component to living better for longer.

## Supplementary Material

fcaf389_Supplementary_Data

## Data Availability

Socio-demographic and imaging data used in this study were taken from the publicly available dataset ‘The Cambridge Centre for Ageing and Neuroscience (Cam-CAN)’ (available at https://cam-can.mrc-cbu.cam.ac.uk/dataset/). The authors are grateful to the scientists involved in the collection of the Cam-CAN dataset and for making it available online. Codes used for machine learning scripts and classification are available on GitHub repository (https://github.com/CCNL-UniPD/brain_age_obesity).

## References

[1] Glatt S, Chayavichitsilp P, Depp C, Schork N, Jeste D. Successful aging: From phenotype to genotype. Biol Psychiatry. 2007;62:282–293. 17210144 10.1016/j.biopsych.2006.09.015

[2] World Health Organization . Obesity and overweight. Accessed 7 May 2025. https://www.who.int/news-room/fact-sheets/detail/obesity-and-overweight

[3] World Health Organization . Obesity and overweight. Accessed 9 June 2021. https://www.who.int/news-room/facts-in-pictures/detail/6-facts-on-obesity

[4] Ng M, Dai X, Cogen RM, et al National-level and state-level prevalence of overweight and obesity among children, adolescents, and adults in the USA, 1990–2021, and forecasts up to 2050. Lancet. 2024;404(10469):2278–2298. 39551059 10.1016/S0140-6736(24)01548-4 PMC11694015

[5] Ng M, Gakidou E, Lo J, et al Global, regional, and national prevalence of adult overweight and obesity, 1990–2021, with forecasts to 2050: A forecasting study for the Global Burden of Disease Study 2021. Lancet. 2025;405(10481):813–838. 40049186 10.1016/S0140-6736(25)00355-1 PMC11920007

[6] Kelly T, Yang W, Chen CS, Reynolds K, He J. Global burden of obesity in 2005 and projections to 2030. Int J Obes. 2008;32(9):1431–1437. 10.1038/ijo.2008.102 18607383

[7] Fernandez-Andujar M, Morales-Garcia E, Garcia-Casares N. Obesity and gray matter volume assessed by neuroimaging: A systematic review. Brain Sci. 2021;11(8):999. 34439618 10.3390/brainsci11080999 PMC8391982

[8] Malenfant J, Batsis J. Obesity in the geriatric population – A global health perspective. J Glob Health Rep. 2019;3:e2019045. 34027129 10.29392/joghr.3.e2019045 PMC8136402

[9] Baker C, Bate A. Briefing paper: Obesity statistics. UK Parliament; 2019.

[10] Ahima RS . Connecting obesity, aging and diabetes. Nat Med. 2009;15(9):996–997. 19734871 10.1038/nm0909-996

[11] Jura M, Kozak L. Obesity and related consequences to ageing. Age (Omaha). 2016;38:23. 10.1007/s11357-016-9884-3 PMC5005878 26846415

[12] Ronan L, Alexander-Bloch AF, Wagstyl K, et al Obesity associated with increased brain age from midlife. Neurobiol Aging. 2016;47:63–70. 27562529 10.1016/j.neurobiolaging.2016.07.010 PMC5082766

[13] Taki Y, Kinomura S, Sato K, et al Relationship between body mass index and gray matter volume in 1,428 healthy individuals. Obesity (Silver Spring). 2008;16(1):119–124. 18223623 10.1038/oby.2007.4

[14] Taki Y, Thyreau B, Kinomura S, et al Correlations among brain gray matter volumes, age, gender, and hemisphere in healthy individuals. PLoS One. 2011;6(7):e22734. 21818377 10.1371/journal.pone.0022734 PMC3144937

[15] Deery HA, Di Paolo R, Moran C, Egan GF, Jamadar SD. The older adult brain is less modular, more integrated, and less efficient at rest: A systematic review of large-scale resting-state functional brain networks in aging. Psychophysiology. 2023;60(1):e14159. 36106762 10.1111/psyp.14159 PMC10909558

[16] Ferreira LK, Busatto GF. Resting-state functional connectivity in normal brain aging. Neurosci Biobehav Rev. 2013;37(3):384–400. 23333262 10.1016/j.neubiorev.2013.01.017

[17] Chan M, Park D, Savalia N, Petersen S, Wig G. Decreased segregation of brain systems across the healthy adult lifespan. Proc Natl Acad Sci U S A. 2014;111:E4997–E5006. 25368199 10.1073/pnas.1415122111 PMC4246293

[18] Jockwitz C, Caspers S. Resting-state networks in the course of aging-differential insights from studies across the lifespan vs. amongst the old. Pflugers Arch. 2021;473(5):793–803. 33576851 10.1007/s00424-021-02520-7 PMC8076139

[19] Zonneveld HI, Pruim RH, Bos D, et al Patterns of functional connectivity in an aging population: The Rotterdam study. Neuroimage. 2019;189:432–444. 30659958 10.1016/j.neuroimage.2019.01.041

[20] Syan S, McIntyre-Wood C, Minuzzi L, Hall G, McCabe R, MacKillop J. Dysregulated resting state functional connectivity and obesity: A systematic review. Neurosci Biobehav Rev. 2021;131:270–292. 34425125 10.1016/j.neubiorev.2021.08.019

[21] Cole JH, Franke K. Predicting age using neuroimaging: Innovative brain ageing biomarkers. Trends Neurosci. 2017;40(12):681–690. 29074032 10.1016/j.tins.2017.10.001

[22] Anaturk M, Kaufmann T, Cole JH, et al Prediction of brain age and cognitive age: Quantifying brain and cognitive maintenance in aging. Hum Brain Mapp. 2021;42(6):1626–1640. 33314530 10.1002/hbm.25316 PMC7978127

[23] de Lange AG, Anaturk M, Rokicki J, et al Mind the gap: Performance metric evaluation in brain-age prediction. Hum Brain Mapp. 2022;43(10):3113–3129. 35312210 10.1002/hbm.25837 PMC9188975

[24] de Lange AG, Anatürk M, Suri S, et al Multimodal brain-age prediction and cardiovascular risk: The whitehall II MRI sub-study. Neuroimage. 2020;222:117292. 32835819 10.1016/j.neuroimage.2020.117292 PMC8121758

[25] Shafto MA, Tyler LK, Dixon M, et al The Cambridge Centre for Ageing and Neuroscience (Cam-CAN) study protocol: A cross-sectional, lifespan, multidisciplinary examination of healthy cognitive ageing. BMC Neurol. 2014;14:204. 25412575 10.1186/s12883-014-0204-1 PMC4219118

[26] Taylor JR, Williams N, Cusack R, et al The Cambridge Centre for Ageing and Neuroscience (Cam-CAN) data repository: Structural and functional MRI, MEG, and cognitive data from a cross-sectional adult lifespan sample. Neuroimage. 2017;144(Pt B):262–269. 26375206 10.1016/j.neuroimage.2015.09.018 PMC5182075

[27] Dye L, Boyle NB, Champ C, Lawton C. The relationship between obesity and cognitive health and decline. Proc Nutr Soc. 2017;76(4):443–454. 28889822 10.1017/S0029665117002014

[28] Molarius A, Seidell JC, Sans S, Tuomilehto J, Kuulasmaa K. Educational level, relative body weight, and changes in their association over 10 years: An international perspective from the WHO MONICA project. Am J Public Health. 2000;90(8):1260–1268. 10937007 10.2105/ajph.90.8.1260 PMC1446346

[29] Chen Y, Lv C, Li X, et al The positive impacts of early-life education on cognition, leisure activity, and brain structure in healthy aging. Aging (Albany NY). 2019;11(14):4923–4942. 31315089 10.18632/aging.102088 PMC6682517

[30] Steffener J . Education and age-related differences in cortical thickness and volume across the lifespan. Neurobiol Aging. 2021;102:102–110. 33765423 10.1016/j.neurobiolaging.2020.10.034 PMC8126642

[31] Zheng H, Cagney K, Choi Y. Predictors of cognitive functioning trajectories among older Americans: A new investigation covering 20 years of age- and non-age-related cognitive change. PLoS One. 2023;18(2):e0281139. 36753483 10.1371/journal.pone.0281139 PMC9907834

[32] Rosenblatt M, Tejavibulya L, Jiang R, Noble S, Scheinost D. Data leakage inflates prediction performance in connectome-based machine learning models. Nat Commun. 2024;15(1):1829. 38418819 10.1038/s41467-024-46150-w PMC10901797

[33] Wareham NJ, Jakes RW, Rennie KL, Mitchell J, Hennings S, Day NE. Validity and repeatability of the EPIC-Norfolk Physical Activity Questionnaire. Int J Epidemiol. 2002;31(1):168–174. 11914316 10.1093/ije/31.1.168

[34] Smith SM, Jenkinson M, Woolrich MW, et al Advances in functional and structural MR image analysis and implementation as FSL. Neuroimage. 2004;23(Suppl 1):S208–S219. 15501092 10.1016/j.neuroimage.2004.07.051

[35] Tournier JD, Smith R, Raffelt D, et al MRtrix3: A fast, flexible and open software framework for medical image processing and visualisation. NeuroImage. 2019;202:116137. 31473352 10.1016/j.neuroimage.2019.116137

[36] Smith SM, Jenkinson M, Johansen-Berg H, et al Tract-based spatial statistics: Voxelwise analysis of multi-subject diffusion data. NeuroImage. 2006;31(4):1487–1505. 16624579 10.1016/j.neuroimage.2006.02.024

[37] Woolrich MW, Ripley BD, Brady M, Smith SM. Temporal autocorrelation in univariate linear modeling of FMRI data. Neuroimage. 2001;14(6):1370–1386. 11707093 10.1006/nimg.2001.0931

[38] Greve DN, Fischl B. Accurate and robust brain image alignment using boundary-based registration. NeuroImage. 2009;48(1):63–72. 19573611 10.1016/j.neuroimage.2009.06.060 PMC2733527

[39] Griffanti L, Salimi-Khorshidi G, Beckmann CF, et al ICA-based artefact removal and accelerated fMRI acquisition for improved resting state network imaging. NeuroImage. 2014;95:232–247. 24657355 10.1016/j.neuroimage.2014.03.034 PMC4154346

[40] Baldassarre A, Ramsey L, Hacker CL, et al Large-scale changes in network interactions as a physiological signature of spatial neglect. Brain. 2014;137(Pt 12):3267–3283. 25367028 10.1093/brain/awu297 PMC4240302

[41] Greenacre M, Groenen P, Hastie T, Iodice D'Enza A, Markos A, Tuzhilina E. Principal component analysis. Nat Rev Methods Primers. 2022;2:100.

[42] Calesella F, Testolin A, De Filippo De Grazia M, Zorzi M. A comparison of feature extraction methods for prediction of neuropsychological scores from functional connectivity data of stroke patients. Brain Inform. 2021;8(1):8. 33877469 10.1186/s40708-021-00129-1 PMC8058135

[43] Siegel JS, Ramsey LE, Snyder AZ, et al Disruptions of network connectivity predict impairment in multiple behavioral domains after stroke. Proc Natl Acad Sci U S A. 2016;113(30):E4367-76. 27402738 10.1073/pnas.1521083113 PMC4968743

[44] Zou H, Hastie T. Regularization and variable selection via the elastic net. J R Stat Soc Series B Stat Methodol. 2005;67(2):301–320.

[45] Handl L, Jalali A, Scherer M, Eggeling R, Pfeifer N. Weighted elastic net for unsupervised domain adaptation with application to age prediction from DNA methylation data. Bioinformatics. 2019;35(14):i154–i163. 31510704 10.1093/bioinformatics/btz338 PMC6612879

[46] Beheshti I, Nugent S, Potvin O, Duchesne S. Bias-adjustment in neuroimaging-based brain age frameworks: A robust scheme. Neuroimage Clin. 2019;24:102063. 31795063 10.1016/j.nicl.2019.102063 PMC6861562

[47] Bashyam VM, Erus G, Doshi J, et al MRI signatures of brain age and disease over the lifespan based on a deep brain network and 14 468 individuals worldwide. Brain. 2020;143(7):2312–2324. 32591831 10.1093/brain/awaa160 PMC7364766

[48] Peters R . Ageing and the brain. Postgrad Med J. 2006;82(964):84–88. 16461469 10.1136/pgmj.2005.036665 PMC2596698

[49] Divo MJ, Martinez CH, Mannino DM. Ageing and the epidemiology of multimorbidity. Eur Respir J. 2014;44(4):1055–1068. 25142482 10.1183/09031936.00059814 PMC4918092

[50] Siman-Tov T, Bosak N, Sprecher E, et al Early age-related functional connectivity decline in high-order cognitive networks. Front Aging Neurosci. 2016;8:330. 28119599 10.3389/fnagi.2016.00330 PMC5223363

[51] Wang J, Knol MJ, Tiulpin A, et al Gray matter age prediction as a biomarker for risk of dementia. Proc Natl Acad Sci. 2019;116(42):21213–21218. 31575746 10.1073/pnas.1902376116 PMC6800321

[52] DeCarli C, Maillard P, Pase MP, et al Trends in intracranial and cerebral volumes of framingham heart study participants born 1930 to 1970. JAMA Neurol. 2024;81(5):471–480. 38526486 10.1001/jamaneurol.2024.0469 PMC10964161

[53] Cox SR, Bastin ME, Ritchie SJ, et al Brain cortical characteristics of lifetime cognitive ageing. Brain Struct Funct. 2018;223(1):509–518. 28879544 10.1007/s00429-017-1505-0 PMC5772145

[54] Fletcher E, Gavett B, Harvey D, et al Brain volume change and cognitive trajectories in aging. Neuropsychology. 2018;32(4):436–449. 29494196 10.1037/neu0000447 PMC6525569

[55] Garcia-Garcia I, Donica O, Cohen AA, et al Maintaining brain health across the lifespan. Neurosci Biobehav Rev. 2023;153:105365. 37604360 10.1016/j.neubiorev.2023.105365

[56] Bozzali M, Padovani A, Caltagirone C, Borroni B. Regional grey matter loss and brain disconnection across Alzheimer disease evolution. Curr Med Chem. 2011;18(16):2452–2458. 21568913 10.2174/092986711795843263

[57] Raji CA, Ho AJ, Parikshak NN, et al Brain structure and obesity. Hum Brain Mapp. 2010;31(3):353–364. 19662657 10.1002/hbm.20870 PMC2826530

[58] Herrmann MJ, Tesar AK, Beier J, Berg M, Warrings B. Grey matter alterations in obesity: A meta-analysis of whole-brain studies. Obes Rev. 2019;20(3):464–471. 30537231 10.1111/obr.12799

[59] Medic N, Ziauddeen H, Ersche KD, et al Increased body mass index is associated with specific regional alterations in brain structure. Int J Obes (Lond). 2016;40(7):1177–1182. 27089992 10.1038/ijo.2016.42 PMC4936515

[60] Brooks SJ, Benedict C, Burgos J, et al Late-life obesity is associated with smaller global and regional gray matter volumes: A voxel-based morphometric study. Int J Obes (Lond). 2013;37(2):230–236. 22290540 10.1038/ijo.2012.13 PMC3572402

[61] Gunstad J, Paul RH, Cohen RA, et al Relationship between body mass index and brain volume in healthy adults. Int J Neurosci. 2008;118(11):1582–1593. 18853335 10.1080/00207450701392282

[62] Ottino-Gonzalez J, Jurado MA, Garcia-Garcia I, et al Allostatic load and disordered white matter microstructure in overweight adults. Sci Rep. 2018;8(1):15898. 30367110 10.1038/s41598-018-34219-8 PMC6203765

[63] Papageorgiou I, Astrakas LG, Xydis V, et al Abnormalities of brain neural circuits related to obesity: A Diffusion Tensor Imaging study. Magn Reson Imaging. 2017;37:116–121. 27899333 10.1016/j.mri.2016.11.018

[64] Samara A, Murphy T, Strain J, et al Neuroinflammation and white matter alterations in obesity assessed by diffusion basis Spectrum imaging. Front Hum Neurosci. 2019;13:464. 31992978 10.3389/fnhum.2019.00464 PMC6971102

[65] Daoust J, Schaffer J, Zeighami Y, Dagher A, Garcia-Garcia I, Michaud A. White matter integrity differences in obesity: A meta-analysis of diffusion tensor imaging studies. Neurosci Biobehav Rev. 2021;129:133–141. 34284063 10.1016/j.neubiorev.2021.07.020

[66] Ding Y, Ji G, Li G, et al Altered interactions among resting-state networks in individuals with obesity. Obesity (Silver Spring). 2020;28(3):601–608. 32090510 10.1002/oby.22731 PMC7098432

[67] Doucet GE, Rasgon N, McEwen BS, Micali N, Frangou S. Elevated body mass index is associated with increased integration and reduced cohesion of sensory-driven and internally guided resting-state functional brain networks. Cereb Cortex. 2018;28(3):988–997. 28119342 10.1093/cercor/bhx008 PMC6059200

[68] Wei D, Zhuang K, Ai L, et al Structural and functional brain scans from the cross-sectional Southwest University adult lifespan dataset. Sci Data. 2018;5:180134. 30015807 10.1038/sdata.2018.134 PMC6049036

[69] Luo N, Sui J, Abrol A, et al Age-related structural and functional variations in 5,967 individuals across the adult lifespan. Hum Brain Mapp. 2020;41(7):1725–1737. 31876339 10.1002/hbm.24905 PMC7267948

[70] Khanna D, Khanna S, Khanna P, Kahar P, Patel BM. Obesity: A chronic low-grade inflammation and its markers. Cureus. 2022;14(2):e22711. 35386146 10.7759/cureus.22711 PMC8967417

[71] Smith JA, Das A, Ray SK, Banik NL. Role of pro-inflammatory cytokines released from microglia in neurodegenerative diseases. Brain Res Bull. 2012;87(1):10–20. 22024597 10.1016/j.brainresbull.2011.10.004 PMC9827422

[72] Yang J, Ran M, Li H, et al New insight into neurological degeneration: Inflammatory cytokines and blood-brain barrier. Front Mol Neurosci. 2022;15:1013933. 36353359 10.3389/fnmol.2022.1013933 PMC9637688

[73] Hauptmann J, Johann L, Marini F, et al Interleukin-1 promotes autoimmune neuroinflammation by suppressing endothelial heme oxygenase-1 at the blood-brain barrier. Acta Neuropathol. 2020;140(4):549–567. 32651669 10.1007/s00401-020-02187-x PMC7498485

[74] Tong Y, Xu S, Huang L, Chen C. Obesity and insulin resistance: Pathophysiology and treatment. Drug Discov Today. 2022;27(3):822–830. 34767960 10.1016/j.drudis.2021.11.001

[75] Hughes TM, Craft S. The role of insulin in the vascular contributions to age-related dementia. Biochim Biophys Acta. 2016;1862(5):983–991. 26657615 10.1016/j.bbadis.2015.11.013

[76] Mazon JN, de Mello AH, Ferreira GK, Rezin GT. The impact of obesity on neurodegenerative diseases. Life Sci. 2017;182:22–28. 28583368 10.1016/j.lfs.2017.06.002

[77] Parimisetty A, Dorsemans A-C, Awada R, Ravanan P, Diotel N, Lefebvre d’Hellencourt C. Secret talk between adipose tissue and central nervous system via secreted factors—An emerging frontier in the neurodegenerative research. J Neuroinflammation. 2016;13(1):67. 27012931 10.1186/s12974-016-0530-x PMC4806498

[78] Bastien M, Poirier P, Lemieux I, Després J-P. Overview of epidemiology and contribution of obesity to cardiovascular disease. Prog Cardiovasc Dis. 2014;56(4):369–381. 24438728 10.1016/j.pcad.2013.10.016

[79] de la Torre JC . Cardiovascular risk factors promote brain hypoperfusion leading to cognitive decline and dementia. Cardiovasc Psychiatry Neurol. 2012;2012:367516. 23243502 10.1155/2012/367516 PMC3518077

[80] Devaux M, Sassi F. Social disparities in hazardous alcohol use: Self-report bias may lead to incorrect estimates. Eur J Public Health. 2016;26(1):129–134. 26585784 10.1093/eurpub/ckv190 PMC4851717

[81] Hofer G, Mraulak V, Grinschgl S, Neubauer AC. Less-intelligent and unaware? Accuracy and unning-Kruger effects for self-estimates of different aspects of intelligence. J Intell. 2022;10(1):10. 35225925 10.3390/jintelligence10010010 PMC8883889

[82] Dohm-Hansen S, English JA, Lavelle A, Fitzsimons CP, Lucassen PJ, Nolan YM. The ‘middle-aging’ brain. Trends Neurosci. 2024;47(4):259–272. 38508906 10.1016/j.tins.2024.02.001

[83] Li G, Hu Y, Zhang W, et al Brain functional and structural magnetic resonance imaging of obesity and weight loss interventions. Mol Psychiatry. 2023;28(4):1466–1479. doi:10.1038/s41380-023-02025-y 36918706 PMC10208984

