# Supplementary material for: Obesity accelerates brain ageing: a multimodal imaging study
Source: Brain Commun. 2025 Oct 13;7(6):fcaf389. doi: 10.1093/braincomms/fcaf389 (PMC12598755; doi:10.1093/braincomms/fcaf389)
Supplement: fcaf389_Supplementary_Data [file fcaf389_supplementary_data.docx]

**Supplementary Table 1.**  Sociodemographic features of normal-weighted (NW) and obese (O) individuals included in the Cam-CAN study. Values denote mean (± SD), numbers of subjects or [percentage of available data]. T-test and Chi-Square were used to compare continuous and categorical variables of the two study groups. Education level expressed as a categorical variable based on qualification level: no degree/O-GCSE levels or equivalent/A levels or equivalent/NVQ, HND, HNC or other professional qualification/CSE or university. PAEE = physical activity energy expenditure. Significant differences between the two study groups are highlighted in bold.

|  | NW (N=261) | O (N=91) | *p* |
| --- | --- | --- | --- |
| *Socio-demographics* |  |  |  |
| Age, years | 50.58 (±18.02) | 60.94 (±16.11) | **<0.001** |
| Educational, qualification | 9/10/12/37/193 | 12/8/7/21/43 | **<0.001** |
| Sex (N, % female) | 125 (48%) | 38 (42%) | 0.37 |
| Handedness | 76.74 (±52.32) | 84.79 (±40.69) | *0.06* |
| PAEE | 45.32 (±21.32) [89%] | 39.79 (±21.97) [86%] | *0.05* |
| *Cognitive scores* |  |  |  |
| MMSE | 29.01 (±1.27) | 28.37 (±1.46) | **<0.001** |
| Verbal fluency | 17.91 (±5.46) | 17.24 (±5.66) | 0.16 |
| Verbal category | 24.79 (±6.59) | 22.95 (±7.32) | **0.01** |
| *Brain features* |  |  |  |
| Whole brain volume | 1638.41 (±161.49) | 1608.41 (±141.50) | 0.11 |
